# Leptin Activates RhoA/ROCK Pathway to Induce Cytoskeleton Remodeling in Nucleus Pulposus Cells

**DOI:** 10.3390/ijms15011176

**Published:** 2014-01-16

**Authors:** Zheng Li, Jinqian Liang, William Ka Kei Wu, Xin Yu, Jun Yu, Xisheng Weng, Jianxiong Shen

**Affiliations:** 1 Department of Orthopaedic Surgery, Peking Union Medical College Hospital, Peking Union Medical College, Beijing 100730, China; E-Mails: kleeo@163.com (Z.L.); string218@126.com (J.L); yuxinbetty@163.com (X.Y.); xshweng@medmail.com.cn (X.W.); 2 Institute of Digestive Disease and State Key Laboratory of Digestive Diseases, Department of Medicine and Therapeutics and LKS Institute of Health Science, The Chinese University of Hong Kong, Hong Kong, China; E-Mails: wukk1980@gmail.com (W.K.K.W.); junyu@cuhk.edu.hk (J.Y.)

**Keywords:** disc degeneration, fluorescent resonance energy transfer (FRET), cytoskeleton, leptin, F-actin

## Abstract

Hyperleptinemia is implicated in obesity-associated lumbar disc degeneration. Nevertheless, the effect of leptin on the intracellular signaling of nucleus pulposus cells is not clear. The current study sought to delineate the possible involvement of the RhoA/ROCK pathway in leptin-mediated cytoskeleton reorganization in nucleus pulposus cells. Nucleus pulposus cells isolated from scoliosis patients were treated with 10 ng/mL of leptin. Fluorescent resonance energy transfer analysis was used to determine the activation of RhoA signaling in nucleus pulposus cells. The protein expression of LIMK1 and cofilin-2 were analyzed by western blot analysis. F-actin cytoskeletal reorganization was assessed by rhodamine-conjugated phalloidin immunoprecipitation. Leptin induced F-actin reorganization and stress fiber formation in nucleus pulposus cells, accompanied by localized RhoA activation and phosphorylation of LIMK1 and cofilin. The RhoA inhibitor C3 exoenzyme or the ROCK inhibitor Y-27632 potently attenuated the effects of leptin on F-actin reorganization and stress fiber formation. Both inhibitors also prevented leptin-induced phosphorylation of LIMK1 and cofilin-2. Our study demonstrated that leptin activated the RhoA/ROCK/LIMK/cofilin-2 cascade to induce cytoskeleton reorganization in nucleus pulposus cells. These findings may provide novel insights into the pathogenic mechanism of obesity-associated lumbar disc degeneration.

## 1. Introduction

Obesity, defined as body mass index (BMI) exceeding 30 kg/m^2^, is a serious public health issue. Globally, more than one billion adults and children are overweight [1]. Obesity is associated with increased risks for various morbidities, including cardiovascular diseases, diabetes, osteoarthritis, and spinal diseases [2]. To this end, obesity is an established risk factor for intervertebral disc degeneration, which is considered to be one of the major etiological factors of low back pain [3]. In a cross-sectional study of consecutive patients who underwent surgery for lumbar intervertebral disc herniation, obesity was found to be strongly over-represented [4]. Aside from mechanical stress, increased circulating levels of obesity-associated hormones, such as sex steroids, insulin, insulin-like growth factor 1, and leptin, are potential pathogenic mediators in obesity-associated lumbar disc degeneration (LDD) [5]. The discovery and isolation of the human obesity gene and its protein product, leptin, have furthered our understanding of the role of this peptide in the pathogenesis of obesity-associated diseases [6]. Leptin, a 16-kDa, 167-amino acid peptide, is released mainly by adipocytes. After binding to its receptors in the hypothalamus, leptin induces a complex regulatory response integrating the control of bodyweight and energy expenditure [7]. In animal experiments, levels of leptin in the blood correlate directly with body fat content. Indeed, in humans, serum leptin levels correlate closely with body mass and may increase from 1–3 ng/mL in non-obese subjects to as high as 100 ng/mL in obese individuals [8].

Several recent studies have indicated that leptin could act as a mitogenic factor in various physiological and pathological contexts, such as lipid homeostasis, insulin secretion, reproductive functions, thermogenesis, angiogenesis and tumorigenesis. Evidence also points to the involvement of leptin in the pathogenesis of LDD. Gruber *et al.* [9] first demonstrated the presence of intracellular leptin and its receptor in annulus cells of the human intervertebral disc. Zhao *et al.* [10] also showed that leptin and leptin receptor-positive cells are commonly seen in proliferating fibrocarilaginous areas. Our previous studies showed that nucleus pulposus (NP) cells expressed leptin receptors and leptin could stimulate the proliferation of disc cells *in vitro* via JAK/STAT, PI3K/Akt and MEK/ERK pathways [11,12]. Although leptin has been shown to affect a number of signal transduction pathways, its signaling mechanism in NP cells remains unclear.

Previous studies have shown that altered expression and organization of cytoskeletal element proteins in NP cells modulate the cellular response to mechanical signals, which is implicated in the development of LDD [13]. In this regard, modulation of cytoskeleton remodeling by leptin has been reported in various cell types. For instance, leptin increases F-actin stress fiber formation in cardiac fibroblasts [14]. Our previous study also showed that leptin could trigger cytoskeletal reorganization in chondrocytes [15]. One of the major signaling pathways regulating the remodeling of the actin cytoskeleton is the small G protein Ras homolog gene family (Rho)/Rho-associated coiled-coil-forming protein kinase (ROCK) pathway [16]. Activated Rho/ROCK mediates intracellular signals through phosphorylating the actin-depolymerizing factor cofilin, which leads to the loss of its ability to sever F-actin [17]. On the other hand, leptin has been shown to induce Rho/ROCK signaling. For instance, the RhoA/ROCK pathway plays a critical role in leptin-induced cardiomyocyte hypertrophy and vascular smooth muscle hypertrophy [18]. Leptin has also been reported to activate the RhoA pathway in kidney epithelial cells, hepatic stellate cells, coronary artery endothelial cells, and colon cancer cells. We hypothesized that the RhoA/ROCK pathway may mediate the enhancing effect of leptin on cytoskeletal remodeling in NP cells. The aim of the present study is therefore to elucidate the relationship among leptin, cytoskeletal remodeling, and RhoA/ROCK signaling in NP cells. Findings of this study will provide novel insights into the effect of obesity on the biochemical and morphological properties of NP cells in healthy and diseased disc tissues.

## 2. Results and Discussion

### 2.1. Leptin Activated the RhoA Signaling in NP Cells

**Figure 1.**
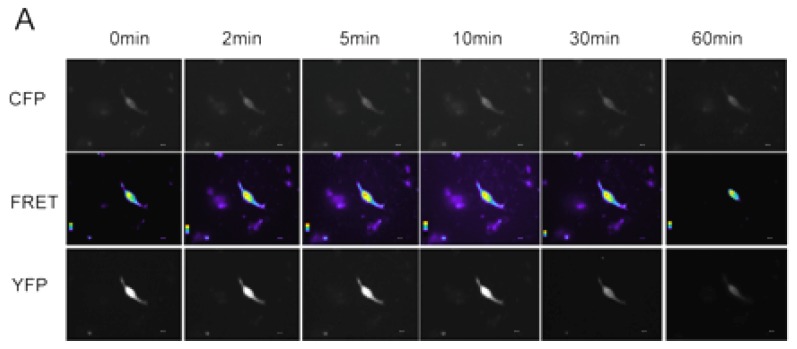

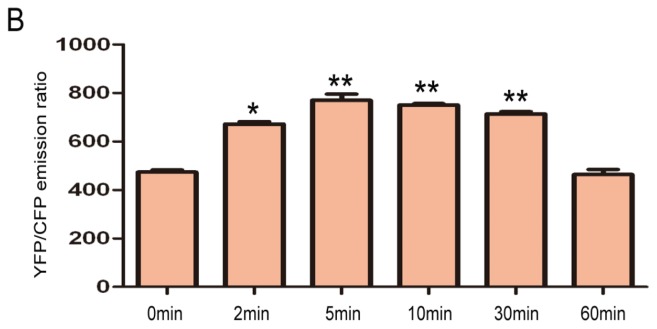
Leptin activated RhoA in human NP cells. (**A**) Human NP cells expressing pRaichu-1237 were imaged as in this figure. The corrected FRET images were pseudo-colored to visualize the localization of active RhoA and FRET intensity. In the color scale, red represents a high FRET signal and blue represents a low signal; (**B**) The YFP/CFP emission ratio was measured by taking the mean intensity of a ROI of the corrected FRET image, divided by the mean intensity of the same region on the CFP image. An average of one cell was quantified for each time point and each image is representative of multiple cells. For each experiment, human NP cells expressing pRaichu 1237× were imaged from 18 h post-transfection. * *p* < 0.05; ** *p* < 0.01, values obtained in the presence of leptin (10 ng/mL) *versus* control (0 min). Scale bars represent 10 μm. Error bars represent SEM.

The RhoA signaling pathway plays a crucial role in the regulation of cytoskeletal reorganization in many cells. We therefore determined if leptin could affect RhoA signaling in NP cells. Without leptin stimulation, constitutive activity of RhoA could be observed in the perinuclear region of NP cells. Upon leptin stimulation, a substantial increase in RhoA activity localized at one end of the cell was observed from 2 to 30 min post-stimulation. The peak induction was observed at 5 min, when RhoA activity was increased by 62%. RhoA activity subsequently returned to basal levels at 60 min (Figure 1). These findings suggest that leptin induced a temporal and localized activation in NP cells.

### 2.2. Leptin Increased Phosphorylation of LIMK1 and Cofilin-2

**Figure 2.**
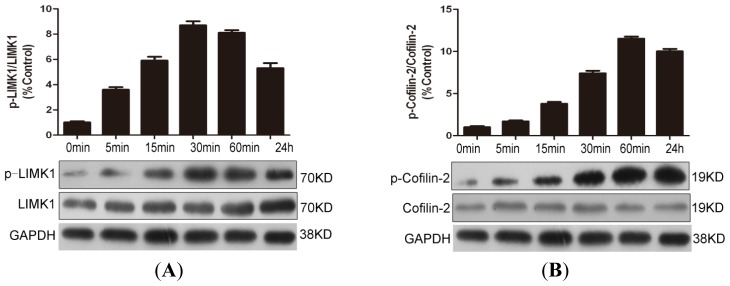
Leptin phosphorylated LIMK1 and confilin-2 in NP cells. After 1-day serum deprivation, leptin (10 ng/mL) was added into the serum-free medium of NP cells for 5 min, 15 min, 30 min, 60 min and 24 h, and then the protein amounts of phosphorylated forms of LIMK1 (p-LIMK1) (**A**) and confilin-2 (p-confilin-2) (**B**) were detected with Western blotting analysis. GAPDH was also detected as a loading control. The signal in each lane was quantified using ImageJ software (v. 2.1.4.7; National Institutes of Health, Boston, MA, USA) and the ratios of p-LIMK1/LIMK1 and p-confilin-2/confilin-2 to GAPDH were determined.

Activated RhoA/ROCK is known to phosphorylate the effector molecules LIMK1 and cofilin-2 to mediate its biological effect. As shown in Figure 2, leptin stimulation time-dependently increased the phosphorylation of LIMK1 and cofilin-2 without altering the total protein levels. The induction of LIMK1 and cofilin-2 phosphorylation could be observed as early as 5 min after leptin stimulation and the maximal stimulation occurred at 30 and 60 min post-stimulation. These findings indicate that leptin could readily activate LIMK/cofilin pathways in human NP cells.

### 2.3. Leptin-Induced Cytoskeletal Reorganization in NP Cells

**Figure 3.**
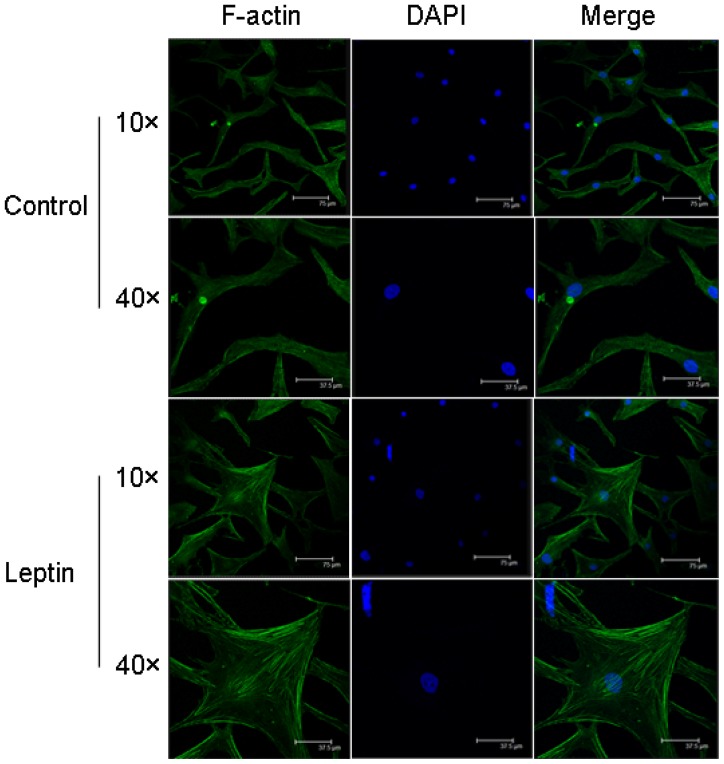
Fluorescence microscopy images showing arrangement of rhodamine-phalloidin-stained (**green**) F-actin filaments in primary human NP cells treated without or with leptin (10 ng/mL). Nuclei were stained with DAPI, shown in **blue**. Images were acquired using laser scanning confocal microscopy under a 10× and 40× objectives. Control NP cells exhibited diffuse cytoplasmic and perinuclear staining of F-actin while leptin-stimulated NP cells showed strong cytoplasmic filamentous structures. Scale bars represent 75 or 37.5 μm.

We first investigated whether leptin could modulate F-actin reorganization in NP cells. F-actin filaments of unstimulated NP cells were predominately localized beneath the cell membrane and exhibited a weak cytoplasmic and perinuclear staining. By contrast, leptin stimulation induced a significant change of cell shape. In this regard, NP cells stimulated with 10 ng/mL leptin for 24 h exhibited epithelioid morphology with increased cellular spreading. F-actin in leptin-stimulated NP cells also showed more intense cytoplasmic staining with occasional localization along filamentous structures (Figure 3).

### 2.4. RhoA and ROCK Inhibition Prevented Leptin-Induced Phosphorylation of LIMK1 and Cofilin-2

**Figure 4.**
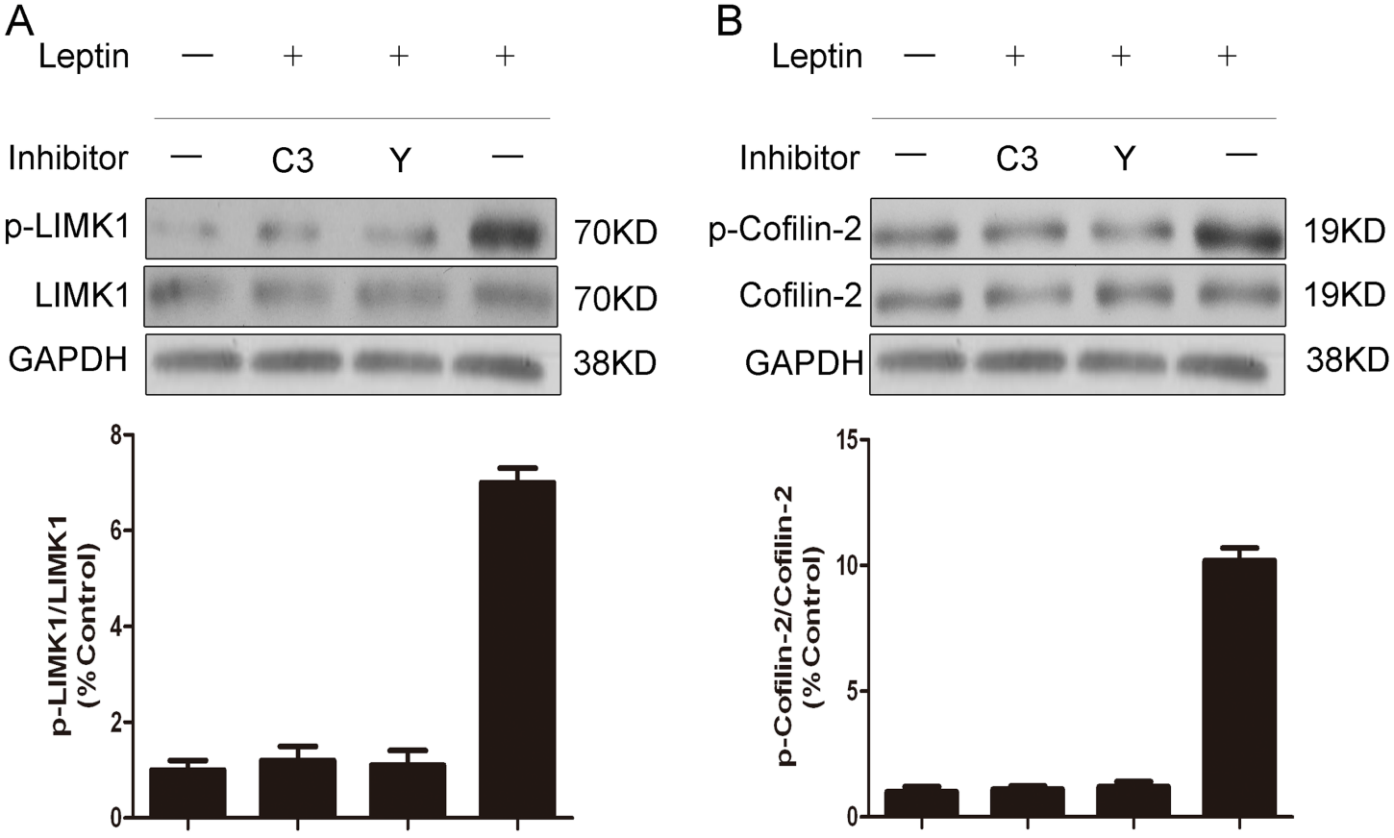
Pharmacological inhibitors of Rho and ROCK prevented phosphorylation of LIMK1 and confilin-2 induced by leptin. NP cells were serum-starved for 24 h, and then pre-treated with C3 (C3 exoenzyme) or Y (Y-276320) for 30 min. The cells were then treated with vehicle (−) or 10 ng/mL leptin (+) in serum-free media for another 30 min. the protein amounts of phosphorylated forms of LIMK1 (p-LIMK1) (**A**) and confilin-2 (p-confilin-2) (**B**) were detected with Western blotting analysis. GAPDH was also detected as a loading control. The signal in each lane was quantified using ImageJ software (v. 2.1.4.7; National Institutes of Health, Boston, MA, USA) and the ratios of p-LIMK1/LIMK1 and p-confilin-2/confilin-2 to GAPDH were determined.

We next examined whether Rho and ROCK inhibition could modulate the phosphorylation of LIMK1 and cofilin-2. NP cells were serum-starved for 24 h, followed by 30-min pre-treatment of C3 exoenzyme (Alexis Biochemicals, Carlsbad, CA, USA), or Y-27632 (Sigma-Aldrich, Oakville, ON, Canada), and then treated with vehicle (−) or 10 ng/mL leptin (+) in serum-free media for another 30 min. Both C3 exoenzyme and Y-27632 completely prevented leptin-induced LIMK1 and cofilin-2 phosphorylation when measured 30 min after leptin administration (Figure 4).

### 2.5. Pharmacological Inhibition of RhoA/ROCK Prevented Leptin-Induced F-Actin Remodeling in NP Cells

**Figure 5.**
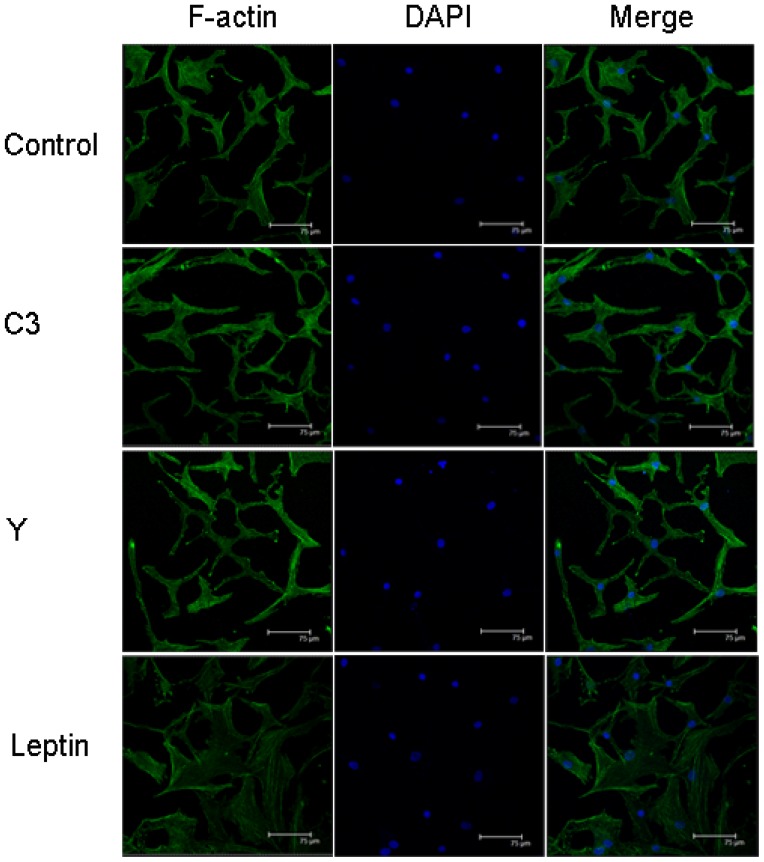
Pharmacological inhibitors of Rho and ROCK prevented F-actin remodeling in human NP cells treated with leptin. NP cells were treated with vehicle (control), specific C3 exoenzyme (C3), or Y-276320 (Y) and incubated in the presence or absence of leptin (10 ng/mL). Fluorescence microscopy images showing both C3 exoenzyme and Y-27632 inhibited leptin-induced actin cytoskeletal reorganization and actin stress fiber formation. Scale bars represent 75 μm.

To assess whether leptin-induced RhoA/ROCK signaling could regulate actin remodeling, NP cells were treated with specific pharmacological inhibitors of Rho and ROCK, followed by 24 h of leptin stimulation. F-actin was analyzed by confocal microscopy. As shown in Figure 5, both C3 exoenzyme and Y-27632 inhibited leptin-induced actin cytoskeletal reorganization and actin stress fiber formation.

### 2.6. Discussion

Given the increasing prevalence of obesity and its association with the development of LDD, understanding the underlying pathogenic mechanism is crucial. The contribution of both mechanical and systemic factors has been suggested. On the one hand, obesity directly increases the mechanical load on the spine. On the other hand, obese people have elevated serum levels of leptin, a known marker of inflammation that is closely associated with cancer and osteoarthritis [19]. The cellular and molecular mechanisms of obesity-related LDD, however, remain unclear. In this study, we investigated the role of RhoA/ROCK signaling in mediating the effect of leptin on cytoskeleton remodeling in primary human NP cells. Leptin activated the RhoA/ROCK/LIMK/confilin pathway to induce cytoskeletal reorganization in NP cells as evidenced by increased RhoA activity, enhanced phosphorylation of LIMK2 and confilin-2 and F-actin stress fiber formation in NP cells. Moreover, the downstream effects were abolished by the RhoA inhibitor C3 or the ROCK inhibitor Y-27632. It has previously been hypothesized that the cytoskeleton, can act as a transducer of mechanical signals in tissues including IVD, bone and articular cartilage. These results supported that leptin might exert a modulatory effect on NP cells cytoskeleton remodeling and thereby contributing to the pathogenesis of LDD.

RhoA/ROCK signaling is an important regulator of many cellular processes, including cellular contraction, motility, morphology, polarity, cell division, and gene expression [20]. Abnormal activation of the Rho/Rho-kinase pathway has been shown to play a role in diseases such as pulmonary hypertension, vasospasm, nerve injury, glaucoma, and osteoarthritis. RhoA is a small guanosine triphosphate (GTP)-binding protein that is activated in response to stimulation of many G protein-coupled receptors. In this study, a FRET-based method was used to examine the spatial and temporal activation of Rho GTPases in live NP cells. A direct correction between FRET efficiency of the probes used in this study and GTP-loading of RhoA has been established and its use in monitoring the balance between GEF and GAP activity for RhoA has been validated [21]. A major advantage of using the probes for measuring RhoA activity is that it allows real-time and quantitative imaging of single live cells. The changes of RhoA activity of an individual cell upon stimulation could be normalized with its basal activity and thus minimized cell-to-cell variations. Furthermore, NP cells used in this study exhibited low growth rate and may undergo dedifferentiation after 3–4 passages. These properties prohibited us from using conventional RhoA activation assay kits, which required large amount of cells. Therefore, FRET represents a superb option for measuring RhoA activity in our study. In particular, the development of fluorescent proteins for FRET microscopy is providing an important tool for monitoring dynamic protein interactions inside live cells. This FRET-based method contributes directly to our finding that leptin could activate RhoA in NP cells [15].

Previous studies have shown that actin cytoskeleton rearrangement induced by ROCK activation requires a cyclic activation and inactivation of cofilin, which is regulated by LIMK-dependent phosphorylation [22]. Our data indicates that leptin rapidly activates Rho GTPases, leading to LIMK1 and cofilin phosphorylation. Importantly, changes of cell shape and actin stress fiber were observed in NP cells. To elucidate the relationship among RhoA activation, LIMK1 and cofilin phosphorylation and F-actin reorganization, we determined the effect of chemical inhibitors of the RhoA/ROCK pathway on leptin-induced F-actin remodeling. C3 (a RhoA inhibitor) and Y-27632 (a ROCK inhibitor) significantly blocked the phosphorylation of LIMK2 and confilin-2 and the F-actin remodeling caused by leptin. These data suggest that the RhoA/ROCK pathway was involved in leptin-induced F-actin remodeling in NP cells.

Actin microfilaments are involved in many cellular processes including alteration of cell shape, movement of organelles, cell migration and adhesion, endocytosis, secretion, contractile ring formation and extracellular matrix assembly [23]. Previous studies have also demonstrated that the cytoskeleton might play a crucial role in mechanotransduction between IVD cells and their surrounding extracellular matrix [24,25]. As the F-actin cytoskeleton plays such a fundamental role in mechanotransduction between the extracellular matrix and IVD cells, it is likely that rearrangement of cytoskeletal networks may promote an imbalance in IVD homoeostasis, favoring a catabolic phenotype which is characteristic of degenerative disc disease [26]. More importantly, cytoskeleton rearrangement has been observed in discs from patients with low back pain or scoliosis compared to discs from healthy subjects [27]. These findings suggest that there is a direct link between cytoskeletal remodeling in NP cells and the development of LDD. In our study, F-actin remodeling was observed in leptin-induced NP cells. It has been suggested that the phenotypic stability of NP cells is critical and alteration of NP cell shape may impede the normal functions of the disc. Our study shows that Rho/ROCK signaling could be activated by leptin in NP cells, accompanied by the change of cell shape. Moreover, this effect was abolished by C3 exoenzyme or Y-27632. However, several limitations of this study have to be taken into consideration, including the lack of age-matched non-degenerative discs as control and the unproven extrapolation of results from LDD patients-derived NP cells to *in vivo* scenario. In addition, the relatively small sample size and the young age (30 to 40 years old) of the patients may limit the interpretation of our findings.

## 3. Experimental Section

### 3.1. Ethics Statement

All of the experimental protocols were approved by the Clinical Research Ethics Committee of the Peking Union Medical College Hospital (Beijing, China). Human lumbar IVD samples obtained from patients undergoing discectomy following approval from the Clinical Research Ethics Committee of the Peking Union Medical College Hospital (Beijing, China) with fully informed written consent of patients.

### 3.2. Nucleus Pulposus Cell Isolation and Culture

NP cells (4 female, 3 male, mean ages: 35 ± 2.5 years; age range 30–40 years) were obtained from surgical IVD tissue samples from patients undergoing disc degeneration surgeries (L4/5) using an established method [11,12]. Routine MRI scans of the lumbar spine were taken for these patients before the operation; the degree of disc degeneration was graded from T2-weighted images using a modified Pfirrmann classification. The disc degeneration grade of these patients was 3. Tissues specimens were first washed thrice with PBS. NP was then separated from the AF using a stereotaxic microscope, and cut into pieces (2–3 mm^3^). Afterwards, NP cells were released from the NP tissues by incubation with 0.25 mg/mL type II collagenase (Sigma, St. Louis, MO, USA) for 12 h at 37 °C in Dulbecco’s modified Eagle medium (DMEM; GIBCO, Grand Island, NY, USA). After isolation, NP cells were resuspended in DMEM containing 10% FBS (GIBCO, Grand Island, NY, USA), 100 μg/mL streptomycin (GIBCO, Grand Island, NY, USA), 100 U/mL penicillin (GIBCO, Grand Island, NY, USA) and 1% l-glutamine (GIBCO, Grand Island, NY, USA), and then incubated at 37 °C in a humidified atmosphere with 95% air and 5% CO_2_. The confluent cells were detached by trypsinization (GIBCO, Grand Island, NY, USA), seeded into 35-mm tissue culture dishes in complete culture medium (DMEM supplemented with 10% FBS, 100 μg/mL streptomycin and 100 U/mL penicillin) in a 37 °C, 5% CO_2_ environment. The medium was changed every 2 days. NP cells cultured *in vitro* within 5 days (*i.e.*, the second passage) were used for subsequent experiments.

### 3.3. Basic Structure of Raichu Probes

Raichu probes were developed and donated by Nakamura T. The basic structure of Raichu probes comprises four modules, donor (CFP), acceptor (YFP), a GTPase, and a GTPase-binding domain of its binding partner. In the Raichu probes for the Rho-family, the positions of a GTPase and a GTPase-binding domain are interchanged to obtain a high signal-to-noise ratio. In the inactive GDP-bound form, CFP and YFP in the probe are located remotely from each other, resulting in excitation mostly from CFP. Upon stimulation, GDP on the GTPase is exchanged for GTP to induce the association of active GTP-bound GTPase with the GTPase-binding domain of the effector protein. This intramolecular binding brings CFP in a close proximity to YFP, thereby causing fluorescent resonance energy transfer (FRET). FRET is simultaneously manifested by quenching of the CFP fluorescence and emanation of the YFP fluorescence; therefore, in the Raichu probes, the YFP/CFP ratio is conveniently used as a representation of FRET efficiency. Since the FRET efficiency of a Raichu probe correlates with the GTP/GDP ratio, the activities of Rho GTPases can be estimated from measurements of the YFP/CFP ratio [15].

### 3.4. Nucleus Pulposus Cell Imageing and FRET Microscopy

The method of FRET microscopy was done using an established method [15]. NP cells were plated on laminin-coated 35-mm glass bottom dishes (MaTek Corp, Ashland, MA, USA) to reach approximately 50%–70% confluence on the next day and transfected with the FRET probes with or without Raichu using Human Chondrocyte Nucleofector™ Kit [27,28]. The following amounts of plasmids were used: 0.1 mg pEYFP-N1, 0.1 mg pECFP-C1, and 0.1 mg pRaichu1237×. Transfected cells were cultured for 18 h before imaging. When treated, cells were imaged every 30 s from 18 to 19 h post-transfection. As a negative control, cells expressing CFP constructs alone or YFP constructs alone were imaged 18 h post-transfection, and no FRET signal was observed. Cells were imaged with a Nikon (Tokyo, Japan) TE2000 inverted microscope equipped with a mercury lamp light source (100 mW), Dual-View™ (Optical Insights, Santa Fe, NM, USA), and a SNAP-HQ cooled charge-coupled device camera (Roper Scientific, Trenton, NJ, USA). The excitation control and image acquisition were achieved using MetaMorph version 5.0 software (Universal Imaging, West Chester, PA, USA). We used the following filters (Chroma, Bellows Falls, Boston, MA, USA) for acquiring CFP, YFP, or FRET images (excitation; dichroic; emission): CFP (S430/25 nm; 455dclp; S470/30 nm), YFP (S500/20 nm; Q515lp; S535/30 nm), and FRET (S430/25 nm; 455dclp; S535/30 nm). Binning 2 × 2 mode and 200 ms of integration time were used.

### 3.5. Western Blotting Analysis

NP cells were treated with 10 ng/mL leptin (Sigma-Aldrich, Oakville, ON, Canada) for 5 min, 10 min, 30 min and 24 h, respectively. Untreated cells were used as controls. Cell lysates were subject to SDS-PAGE and transferred to PVDF membranes (Millipore, Boston, MA, USA). Primary antibodies against the following proteins were used: LIMK1, p-LIMK1, cofilin-2, p-cofilin-2 (Abcam, Cambridge, UK) and GAPDH (Abcam, Cambridge, UK). HRP-conjugated secondary antibodies were used. Signal was detected using ECL kit (Millipore, Boston, MA, USA).

### 3.6. Immunofluorescence Microscopy

Coverslips were placed into 24-well plate and then NP cells were plated and treated with 10 ng/mL leptin for 48 h. After incubation and treatment, medium was removed and then cells were washed twice with PBS and fixed with 3.5% formaldehyde for 30 min at 37 °C. The cells were rinsed with PBS for 3 × 2 min, permeabilized with 0.1% Triton X-100 in PBS for 20 min and blocked with 3% BSA and 0.05% Tween 20 in PBS for 30 min at room temperature and then incubated overnight at 4 °C with primary antibody. F-actin was stained with rhodamine-phalloidin (Sigma-Aldrich, Oakville, ON, Canada). Nuclei were stained with 4,6-diamidino-2-phenylindole (DAPI) (Sigma-Aldrich, Oakville, ON, Canada). Fluorescence images were acquired with a Leica TCS SP2 confocal microscopy (Leica, Mannheim, Germany) using the Leica Confocal Sofware (Leica, Mannheim, Germany).

### 3.7. Statistical Analysis

MetaMorph software (v. 6.0; Molecular Devices, Boston, MA, USA) was used to analyze the cell image data. Average background signal was determined as the mean fluorescence intensity from a blank area and was subtracted from the raw image before carrying out FRET calculations. The mean intensity of a ROI of the corrected FRET image was measured. Western blotting results were normalized with GAPDH. The mean and standard deviation were used to express protein levels of ROCK, p-ROCK, cofilin-2, p-cofilin-2, LIMK, and p-LIMK1. Statistical analyses were performed using the SPSS statistical software program (v. 17.0; IBM, Chicago, IL, USA). Data were expressed as means ± S.E.M. Independent experiments were performed thrice. Statistical analysis was performed with student’s *t*-test. *p* values less than 0.05 were considered statistically significant.

## 4. Conclusions

Our study has demonstrated that leptin activated the RhoA/ROCK/LIMK/cofilin pathway and induced cytoskeleton reorganization in NP cells and the downstream effects could be abolished by the inhibition of RhoA or ROCK. It is anticipated that, with a further understanding of the complex biology of LDD and its relationship with obesity, novel therapeutic strategies will emerge.
